# Supplementary material for: Does the choice of nucleotide substitution models matter topologically?
Source: BMC Bioinformatics. 2016 Mar 24;17:143. doi: 10.1186/s12859-016-0985-x (PMC4806516; doi:10.1186/s12859-016-0985-x)
Supplement: Additional file 1 — Supplementary information. (PDF 120 kb) [file 12859_2016_985_MOESM1_ESM.pdf]

RESEARCH

Teaching Supplement for: Does the choice of nucleotide substitution models matter topologically?

Michael Hoff[1†], Stefan Orf[1†], Benedikt Riehm[1†], Diego Darriba[2] and Alexandros Stamatakis[1,2*]

{michael.hoff, steffan.orf, benedikt.riehm}@student.kit.edu
{diego.darriba, alexandros.stamatakis}@h-its.org

Abstract

Background: This on-line supplement describes the teaching aspects of the main paper, since the entire project was carried out by Master level students in the computer science department of the Karlsruhe Institute of Technology. In the following, we cover teaching issues regarding the setup of the course and the practical and summarize student and teacher experiences.

Keywords: phylogenetics; nucleotide substitution; model selection; information criterion; BIC; AIC

1 Teaching Perspective, Goals and Course Outline

Courses at the Master level in our computer science department are organized in so-called modules over two semesters. In the first semester of the Bioinformatics module, we teach a lecture called "Introduction to Bioinformatics for Computer Scientists", since KIT does not offer a Bioinformatics degree. This lecture covers basic topics such as an introduction to molecular biology, pair-wise sequence alignment, BLAST, de novo and by-reference sequence assembly, multiple sequence alignment, phylogenetic inference, MCMC methods, and population genetics.

In the second semester of the module, students can choose if they want to do a seminar presentation or the programming practical whose results we describe here. The goal of the practical is to carry out a self-contained project and write, as well as release software, that is useful to the evolutionary biology community. Another focus is on learning to use tools that enhance software quality. Note that, at a CS department designing "classic" bioinformatics analysis pipelines using scripting languages is typically not considered as "real programming" by the students. Hence, we need to define a project that requires coding in `C/C++` or `Java`. One should also strive to avoid having the students extend existing software, since this is generally frustrating and hinders creativity.

We thus decided to essentially re-implement the paper on Bayesian model selection [1], but in a ML framework. This project allows to apply a broad range of skills acquired in the Bioinformatics and other master-level modules at our department. Initially, students need to read and understand the original paper. Then, they can use their algorithmic knowledge and training to design an algorithm that correctly enumerates all possible time-reversible substitution models. Subsequently, they can use the PLL to carry out the model tests. Note that, implementing the likelihood function efficiently and in a numerically stable way is a tedious task that requires comprehensive background knowledge and experience. This can not be accomplished by students during a single semester. Hence, the PLL lends itself for conducting such practicals because students will learn how to use a scientific high performance library. Thereby, they can also use their knowledge on phylogenetic likelihood models and the AIC,

*Correspondence: Alexandros.Stamatakis@h-its.org
[1]Karlsruhe Institute of Technology, Department of Informatics, Kaiserstraße 12, 76131, Karlsruhe, Germany
[2]The Exelixis Lab, Scientific Computing Group, Heidelberg Institute for Theoretical Studies, Schloss-Wolfsbrunnenweg 35, 69118 Heidelberg, Germany
Full list of author information is available at the end of the article
†Equal contributor

AICc, and BIC criteria presented in the lectures. By requesting students to parallelize the code via a simple master-worker approach they can also deploy their parallel computing knowledge (from other modules) and use MPI (Message Passing Interface) in practice.

Finally, we also want to encourage critical thinking. Thus, we ask the question if such extensive model testing is actually required. In other words, we wanted to assess if using the best model for a ML tree search (as implemented in the PLL) induces substantial differences in the final tree topologies compared to an inference that simply relies on GTR. In these tests the students are also given the opportunity to calculate Robinson-Foulds (RF [2]) distances between trees that were covered in the lectures. Another question we asses is how different ways to incorporate the sample size of the two-dimensional sample (the #taxa and #sites in the multiple sequence alignment) into AIC and BIC criteria affects the model selection process.

In terms of project documentation, students are usually required to write a report. However, in the present case, we jointly took the decision to write a paper about the practical. This has the positive effect that students also learn how to write scientific papers.

2 Teaching Conclusions

In the following we outline our subjective perception of the teaching outcome of this practical from the student and teacher point of view.

Student View

From the student's perspective, the practical was well-organized and always took place in a very constructive, stress-free atmosphere. Prior to the practical, Alexis made sure that the task was feasible by asking one of his lab members to implement a proof-of-concept solution. This way, we were sure that the task at hand is doable. We also had a responsive advisor (Diego Darriba) for asking implementation questions regarding our program and the usage of the PLL.

The main challenge was to become familiar with the PLL. This scientific library has a plethora of features and covers a broad range of different application scenarios. It therefore took a while, until the first model evaluation on a tree was successful. During this time we met Alexis every week to discuss our latest achievements and issues.

The generalization towards testing all models was then finished quickly and we could start with the parallelization of the code. Our main challenge for the parallelization was to decide which data to communicate and how to design the application in an understandable and reusable manner.

Towards the end of the practical we focused on testing the models. We wrote several scripts to automatically executed our program on each test dataset. As soon as the results were calculated, we built scripts to retrieve and visualize the data for this paper.

Teacher View

This was the first programming practical I carried out at KIT. The teaching experience was generally very positive because we worked on something interesting that I had been wondering about since listening to a talk on the topic given by John Huelsenbeck and not on a completely constructed programming exercise. Furthermore, the students were highly motivated and the group was small which allowed for close interactions and good supervision. The students were also very enthusiastic about trying to write a paper instead of a boring report, despite the fact that this induced a considerable amount of extra work that extended well into the following semester.

Thus, we will continue running the practical based on this scheme. This semester's task is to implement a numerically stable and highly optimized (using SSE3 and AVX vector intrinsics as well as cache optimization techniques) version of the TKF91 [3] statistical alignment kernel from scratch.

Competing interests

The authors declare that they have no competing interests.

Author's contributions

M.H., S.O., and B.R. (the students) implemented the tool and conducted the experiments. A.S. taught the course and designed as well as supervised the programming task. D.D. provided support, including bug fixes, for the PLL and also helped with the information criteria. All authors contributed to drafting and writing the manuscript.

Acknowledgments

We wish to thank John Huelsenbeck for providing the test datasets from the original paper, Andre J. Aberer for providing additional test datasets, and Lucas Czech for implementing an initial prototype version for the task to verify if it was 'doable'.

Author details

[1]Karlsruhe Institute of Technology, Department of Informatics, Kaiserstraße 12, 76131, Karlsruhe, Germany. [2]The Exelixis Lab, Scientific Computing Group, Heidelberg Institute for Theoretical Studies, Schloss-Wolfsbrunnenweg 35, 69118 Heidelberg, Germany.

References

1. Huelsenbeck, J.P., Larget, B., Alfaro, M.E.: Bayesian phylogenetic model selection using reversible jump markov chain monte carlo. Molecular Biology and Evolution **21**(6), 1123–1133 (2004). doi:10.1093/molbev/msh123. http://mbe.oxfordjournals.org/content/21/6/1123.full.pdf+html
2. Robinson, D., Foulds, L.R.: Comparison of phylogenetic trees. Mathematical Biosciences **53**(1), 131–147 (1981)
3. Thorne, J.L., Kishino, H., Felsenstein, J.: An evolutionary model for maximum likelihood alignment of dna sequences. Journal of Molecular Evolution **33**(2), 114–124 (1991)
